# Visual performance of a non-diffractive extended depth of focus IOL in stable early-stage Keratoconus: prospective pilot study

**DOI:** 10.1007/s10792-026-04186-5

**Published:** 2026-08-04

**Authors:** Erika Bonacci, Camilla Pagnacco, Adriano Fasolo, Francesca Barzaghi, Ariel Bonato, Ismaila Idrizi, Marco Anastasi, Emilio Pedrotti

**Affiliations:** 1 Ophthalmology Clinic, Department of Engineering for Innovation Medicine, University of Verona, Verona, Italy; 2 Ophthalmology Clinic, Department of Surgery, Dentistry, Maternity and Infant, University of Verona, Verona, Italy; 3 Research Unit, The Veneto Eye Bank Foundation, Venice, Italy

**Keywords:** Premium IOL, Keratoconus, Early-stage Keratoconus, Non-diffractive EDOF IOL, Visual outcomes

## Abstract

**Aims:**

To evaluate visual performance, contrast sensitivity (CS), optical quality, and subjective satisfaction of Vivity extended depth-of-focus (EDOF) premium intraocular lens (pIOL) in early keratoconus (KC).

**Methods:**

In this prospective, interventional, single-center clinical study, ten patients with mild bilateral KC and cataract received AcrySof IQ Vivity pIOL. Pre- and postoperative assessments included monocular and binocular uncorrected and distance-corrected visual acuities at 4 m (UDVA, DCVA), uncorrected and distance-corrected intermediate and near visual acuities at 66 cm (UI66VA, DCI66VA) and 40 cm (UNVA, DCNVA), defocus curve, CS, wavefront aberrometry (RMS, PSF, Strehl ratio), halometry, and subjective outcomes.

**Results:**

At 3 months, mean ± SD monocular DCVA in the dominant eyes (primary endpoint) was 0.06 ± 0.13 logMAR. A statistically significant improvement was observed between preoperative and postoperative monocular and binocular UDVA, UNVA, DCNVA (*p* < 0.001), and DCVA (*p* = 0.03). Binocular DCI66VA and UI66VA were 0.10 ± 0.15 and 0.14 ± 0.16 logMAR, respectively. The defocus curve was ≤ 0.20 logMAR from + 0.50 D to – 2.00 D. CS peaked under scotopic conditions at 6 cpd, total ocular RMS improved from 2.15 ± 1.98 to 1.09 ± 0.77 (*p* = 0.09). NEI-RQL-42 scores indicated favourable patient-reported outcomes, particularly for glare, clarity of vision, and satisfaction with correction.

**Conclusion:**

In this small prospective pilot study, bilateral implantation of a non-diffractive EDOF IOL was associated with favourable short-term visual outcomes in highly selected patients with stable early keratoconus. These preliminary findings suggest that early stable KC may not represent an absolute contraindication to non-diffractive EDOF IOL implantation, but larger comparative studies with longer follow-up are required.

**Supplementary Information:**

The online version contains supplementary material available at 10.1007/s10792-026-04186-5.

## Introduction

Keratoconus (KC) is a progressive, non-inflammatory corneal ectasia characterized by stromal thinning and irregular astigmatism, often resulting in significant high-order aberrations (HOAs) that impair visual quality. Due to these optical limitations, the use of premium intraocular lenses (pIOLs) has traditionally been considered contraindicated in these patients [1]. However, in early-stage KC, corneal shape and optical quality may remain acceptable, with HOAs within tolerable limits [2–4], making carefully selected pIOLs a potential option.

Among pIOLs, extended-depth-of-focus (EDOF) pIOLs provide significantly fewer visual disturbances than multifocal lenses [5–8] and, as they are designed to maintain functional retinal image quality across an extended focal range [9], can offer greater tolerance to minor defocus and wavefront aberrations, possibly mitigating HOAs and coma-like distortions characteristic of early KC. This is especially true for non-diffractive EDOF pIOLs, which preserve high level of contrast sensitivity (CS) by not splitting light energy into multiple focal points [10], like the AcrySof IQ Vivity pIOL (Alcon, Fort Worth, TX, USA) which uses wavefront-shaping technology to elongate the focal range [11, 12] with reported favourable outcomes in patients with challenging ocular conditions such as glaucoma, macular degeneration and epiretinal membrane [13–15].

The present pilot study aims to evaluate the visual performance, tolerance, and optical quality outcomes after Vivity pIOL implantation in patients with cataract and stable, early-stage KC. This investigation explores the clinical feasibility of pIOLs in a population traditionally excluded from such refractive options.

## Materials and methods

This prospective, interventional, single-centre pilot study included patients with cataract with mild KC evaluated with MS-39 anterior segment (AS) OCT (CSO, Florence, Italy) which combines Placido-disc corneal topography with spectral-domain anterior segment OCT and provides anterior and posterior corneal curvature, pachymetric mapping, epithelial thickness mapping, and aberrometric assessment. The study was approved by the local ethics committee (prot. n. 22,546), registered on clinicaltrials.gov (NCT07338708), and adhered to the Declaration of Helsinki.

### Inclusion criteria

Patients were eligible if they required bilateral cataract surgery and had a diagnosis of bilateral KC established on the basis of clinical examination and MS-39 anterior segment OCT/topography assessment.

KC diagnosis was based on the overall clinical and tomographic pattern, including corneal morphology, anterior and posterior elevation, pachymetric profile, topographic asymmetry, and exclusion of other corneal disorders. The ABCD grading output available within the MS-39 software was used to describe KC severity, rather than as a standalone diagnostic criterion [4, 16].

Only patients with stable mild bilateral KC were included. Mild KC was defined as a maximum grade of 1 in each individual ABCD parameter (A, B, C, and D) according to the MS-39 software-derived ABCD output. Therefore, only eyes graded A0–A1, B0–B1, C0–C1, and D0–D1 were eligible [17].

KC stability was defined as a maximum keratometric change < 1.0 D and a maximum pachymetric reduction < 2% over the preceding 5 years [18], together with documented tomographic stability over time. Additional eligibility criteria included absence of corneal scarring and documented historical best spectacle-corrected distance visual acuity of 0.1 to 0.0 logMAR before cataract-related visual decline. Historical best-corrected visual acuity was obtained from ophthalmological records documenting visual function before cataract onset and was distinct from the preoperative visual acuity measured at enrolment, which reflected cataract-related impairment superimposed on the underlying KC.

Patients were also required to agree to attend all scheduled follow-up visits, undergo second-eye surgery within 7 days after the first-eye procedure, and provide written informed consent.

### Exclusion criteria

Patients with any ABCD parameter greater than grade 1 on the MS-39 software-derived ABCD output were excluded. Furthermore, patients who had any ocular comorbidity other than cataract and keratoconus that could potentially affect visual outcomes, including glaucoma, retinal disease, amblyopia, pseudoexfoliation syndrome, zonular laxity, or a history of uveitis were excluded. Additional exclusion criteria were corneal opacity, including any stromal scarring, advanced or progressive keratoconus, and any previous ocular surgery other than corneal cross-linking performed at least 5 years before enrolment. Patients who had undergone corneal cross-linking within 5 years before enrolment were also excluded.

Cylinder magnitude was not used as an independent exclusion criterion. It was considered only for IOL model selection.

### Study protocol

At each study visit, all patients received a comprehensive ophthalmologic evaluation. Preoperative assessment included monocular and binocular uncorrected and corrected visual acuity, both for distance at 4 m, at intermediate 66 cm and near at 40 cm (UDVA, DCVA, UI66VA, DCI66VA, UNVA and DCNVA), using CSO Vision Charts software (version 14.0, CSO); corneal tomography (MS-39, CSO, Florence, Italy); ocular optical quality analysis by Pyramidal WaveFront-based sensor aberrometer (Osiris T Aberrometer, CSO, Florence, Italy); optical biometry (Lenstar 900, Haag-Streit Diagnostics, Koeniz, Switzerland); intraocular pressure measurement, anterior segment slit-lamp examination, including cataract grading according to the Lens Opacities Classification System III (LOCS III) [19], dilated fundoscopy, and macular evaluation through spectral-domain OCT (Spectralis OCT, Heidelberg Engineering GmbH, Germany).

Ocular dominance was determined preoperatively using the Miles test [20].

All patients underwent standard phacoemulsification by the same expert surgeon (E.P.) with a 2.2 mm phaco probe under topical anaesthesia. All pIOLs were implanted in the capsular bag targeting emmetropia bilaterally. The Kane KC formula, a version of the Kane formula specifically optimised for eyes with KC, was used for IOL power calculation [21]. Patients were counselled preoperatively that the presence of KC could limit refractive predictability and that mild residual refractive error might persist after surgery.

In eyes with regular corneal astigmatism ≥ 1.00 D, the toric model of the Vivity pIOL was implanted to optimize postoperative refractive outcomes [22].

Complete ophthalmological examination was performed at 3 months follow-up and included: binocular and monocular UDVA and DCVA at 4 m, UNVA and DCNVA at 40 cm, uncorrected and distance-corrected intermediate visual acuity at 66 cm (UI66VA and DCI66VA) by means of CSO Vision Charts V14.0 (CSO, Florence, Italy), binocular defocus curve, monocular and binocular CS testing (cpg) under photopic (80 cd/m^2^), mesopic (6 cd/m^2^), and scotopic (3 cd/m^2^) light conditions (CSV 1000 HGT; Vector Vision, Greenville, OH), ocular optical quality analysis by Pyramidal WaveFront-based sensor aberrometer (Osiris T Aberrometer, CSO, Florence, Italy), binocular and monocular halo test (Aston Halometer), posterior capsular opacity (PCO) evaluation, intra- and postoperative complications and adverse events and the National Eye Institute Refractive Error Quality of Life Instrument 42 (NEI-RQL-42) questionnaire.

Binocular defocus curve was assessed from + 1.50 D to – 3.50 D in 0.50 D increments, relative to the DCVA, by recording the best achievable visual acuity at each defocus step. To minimize learning bias, letter sequences were randomized and patients’ eyes were occluded between lens changes [23].

The NEI- RQL-42 consists of 13 subscales with 42 items in 16 different question/response category formats. Each item is converted to a 0–100 possible range. All items are scored so that high score represents better quality of life [24–27].

Binocular halo perception was evaluated using Aston Halometer, which employs a central LED glare source (Golden Dragon Plus LCW W5AM.PC, 5000 K; Osram Licht AG) displayed on an iPad4 [28, 29].

Toric IOL alignment was assessed on postoperative days 1 and 7 using the Toric IOL Assistant of the Osiris-T Aberrometer. Rotation was considered clinically relevant when the angular deviation exceeded 5° or when residual astigmatism was considered visually significant. Surgical realignment was performed when clinically indicated [30].

The Osiris T Aberrometer was used to assess the preoperative and postoperative (at 3 months) total root mean square (RMS), RMS and PSF Strehl ratio at 3 mm and 4 mm of pupil diameter [31].

In case of PCO grade 3 or above [32], treatment was required by neodymium-doped yttrium aluminium garnet laser-capsulotomy, and the 3 months evaluation was re-performed after 14 days.

Although a non-diffractive EDOF IOL was implanted, the procedure was not framed or managed as standard refractive cataract surgery. Postoperative residual refractive error was managed conservatively. At 3 months, spectacle correction was considered the first-line option in symptomatic patients. Corneal refractive enhancement was not included in the management protocol because of the underlying ectatic disease.

### IOL

The AcrySof® IQ Vivity® (Alcon, Fort Worth, TX, USA) is a single-piece, C-loop haptic, hydrophobic acrylic EDOF pIOL that extends the depth of focus through a non-diffractive wavefront-shaping design rather than light-splitting optics.

The lens design is based on a monofocal aspheric platform with an aberration-neutral anterior surface. The central optical zone incorporates a X-WAVE™ technology that stretch and shift the wavefront, creating a continuous elongated focal range while maintaining efficient light transmission. Unlike diffractive multifocal IOLs, this design preserves CS and minimizes photic phenomena, making it potentially more tolerant of mild optical imperfections such as the low levels of HOAs present in early-stage KC. The toric version, AcrySof® IQ Vivity® Toric, shares the same non-diffractive EDOF design and offers cylinder powers from 1.03 D to 4.11 D at the IOL plane.

### Statistical analysis

All statistical analyses were performed using STATA software, version 13.0 (StataCorp, College Station, TX, USA). The distribution of continuous variables was assessed using the Shapiro–Wilk test. Normally distributed variables are reported as mean ± standard deviation (SD), while categorical variables are reported as absolute and relative frequencies; when the distribution of paired differences was not normal, the Wilcoxon signed-rank test was used.

The primary endpoint was the DCVA in the dominant eye at the 3 month postoperatively. Binocular outcomes were also analysed and were considered exploratory.

Paired t-tests analyses were conducted between preoperative and postoperative UDVA, DCVA, UI66VA, DCI66VA, UNVA, DCNVA, and Total RMS to assess postoperative functional improvement. When the distribution of paired differences was normal, paired t-tests were used. Intraoperative and postoperative complications and adverse events were summarized as absolute and relative frequencies to evaluate safety.

Because this was a prospective pilot study, the sample size was determined using a precision-based approach rather than a hypothesis-testing power calculation. Based on a previous pilot series reporting low variability in monocular DCVA in eyes with stable KC implanted with a premium IOL [33], 10 subjects, corresponding to 10 dominant eyes, were considered adequate to estimate the primary endpoint with clinically acceptable precision. Assuming an SD of 0.017 logMAR, for postoperative dominant-eye DCVA, a sample size of 10 subjects was estimated to provide a 95% confidence interval half-width of approximately 0.02 logMAR, corresponding to one ETDRS letter. Sample-size calculations were performed using PASS 2023, version 23.0.2.

## Results

Twenty eyes from 10 patients (7 females and 3 males, all Caucasian) with a mean age of 52 ± 4 years (median: 53.5 years, range: 43–56 years) were included. KC stage A0B0C0 was found in 4 eyes (20%), A0B0C1 in 5 eyes (25%), A0B1C1 in 5 eyes (25%), and A1B1C1 in 6 eyes (30%), respectively. Among dominant eyes, 4 were stage A0B0C0 (40%), 3 A0B0C1 (30%), and 3 A0B1C1 (30%).

Based on LOCS III grading, the predominant lens opacity was grade 3 nuclear cataract in 5 patients, grade 4 posterior subcapsular cataract in 3 patients, and grade 4 cortical cataract in 2 patients.

All patients completed the 3 month follow-up visit.

Mean corneal coma RMS and coma-like aberrations were 0.18 ± 0.13 and 0.26 ± 0.22 and 0.16 ± 0.01 and 0.23 ± 0.01 in dominant eyes.

The average spherical IOL dioptric power was 18.9 ± 3.17 D (median: 19.25 D, range: 12 to 23 D), and in the dominant eyes it was 19.2 ± 2.12 D (median: 20.15 D, range: 14 to 22 D).

Fifteen eyes (75%) showed regular corneal cylinder and received a toric EDOF IOL with mean cylindrical correction of 1.75 ± 0.85 D. Eight of the 10 dominant eyes (80%) received the toric version of the IOL with mean cylindrical correction of 1.25 ± 0.70 D. Only one patient showed toric IOL rotation of 11° requiring surgical repositioning.

At 3 months, no PCO was observed and no adverse events occurred.

**Fig. 1:**
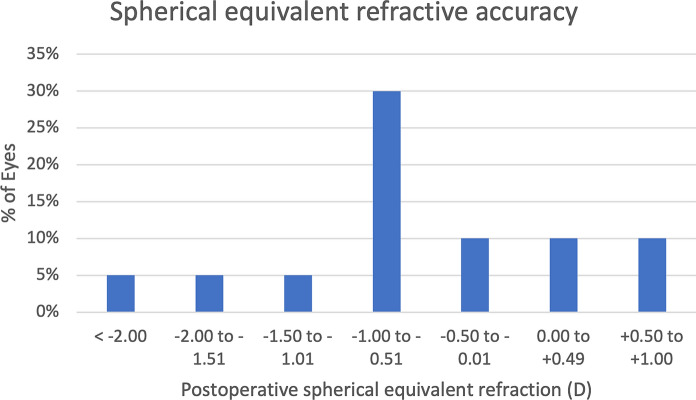
Spherical equivalent refractive accuracy. D, diopters

The mean postoperative manifest refraction spherical equivalent was 0.48 ± 0.69 overall and 0.40 ± 0.92 in dominant eyes at 3 months follow-up. (Fig. 1) The mean preoperative manifest refractive cylinder was 1.75 ± 0.85 D. At 3 months, the mean postoperative residual refractive cylinder was 1.16 ± 0.33 D overall and 1.08 ± 0.34 D in the dominant eyes.

Postoperative VA significantly improved across all tested distances. Monocular UDVA and DCVA improved from 0.64 ± 0.15 to 0.16 ± 0.17 (*p* < 0.001) and from 0.34 ± 0.24 to 0.06 ± 0.08 (*p* < 0.001), respectively. Binocular UDVA and DCVA improved from 0.54 ± 0.15 to 0.08 ± 0.10 (*p* < 0.001) and from 0.23 ± 0.09 to 0.05 ± 0.10 (*p* = 0.003). Monocular and binocular UNVA improved from 0.55 ± 0.14 to 0.33 ± 0.24 (*p* = 0.004) and from 0.41 ± 0.10 to 0.22 ± 0.10 (*p* < 0.001), respectively. DCNVA improved from 0.65 ± 0.34 to 0.29 ± 0.10 monocularly (*p* = 0.002) and from 0.51 ± 0.21 to 0.22 ± 0.23 binocularly (*p* = 0.004). Monocular UI66VA and DCI66VA improved from 0.35 ± 0.21 to 0.13 ± 0.16 logMAR (*p* = 0.004) and from 0.51 ± 0.11 to 0.14 ± 0.06 logMAR (*p* < 0.001), respectively. Binocular UI66VA and DCI66VA improved from 0.30 ± 0.25 to 0.09 ± 0.13 logMAR (*p* < 0.001) and from 0.41 ± 0.22 to 0.10 ± 0.15 logMAR (*p* < 0.001), respectively (Table 1).

**Table 1:** Preoperative and postoperative monocular and binocular visual acuity

Monocular	Pre-operative mean ± SD (LogMAR)	Post-operative mean ± SD (LogMAR)	*P* value
UDVA	0.64 ± 0.15	0.16 ± 0.17	*p* < 0.001
DCVA	0.34 ± 0.24	0.06 ± 0.08	*p* < 0.001
UI66VA	0.35 ± 0.21	0.13 ± 0.16	*p* = 0.004
DCI66VA	0.51 ± 0.11	0.14 ± 0.06	*p* < 0.001
UNVA	0.55 ± 0.14	0.33 ± 0.24	*p* = 0.004
DCNVA	0.65 ± 0.34	0.29 ± 0.10	*p* = 0.002
*Binocular*			
UDVA	0.54 ± 0.15	0.08 ± 0.10	*p* < 0.001
DCVA	0.23 ± 0.09	0.05 ± 0.10	*p* = 0.003
UI66VA	0.30 ± 0.25	0.09 ± 0.13	*p* < 0.001
DCI66VA	0.41 ± 0.22	0.10 ± 0.15	*p* < 0.001
UNVA	0.41 ± 0.10	0.22 ± 0.10	*p* < 0.001
DCNVA	0.51 ± 0.21	0.22 ± 0.23	*p* = 0.004

SD, standard deviation; UDVA, uncorrected distance visual acuity; DCVA, distance-corrected visual acuity; UI66VA, uncorrected intermediate visual acuity at 66 cm; DCI66VA, distance-corrected intermediate visual acuity at 66 cm; UNVA, uncorrected near visual acuity; DCNVA, distance-corrected near visual acuity

**Fig. 2:**
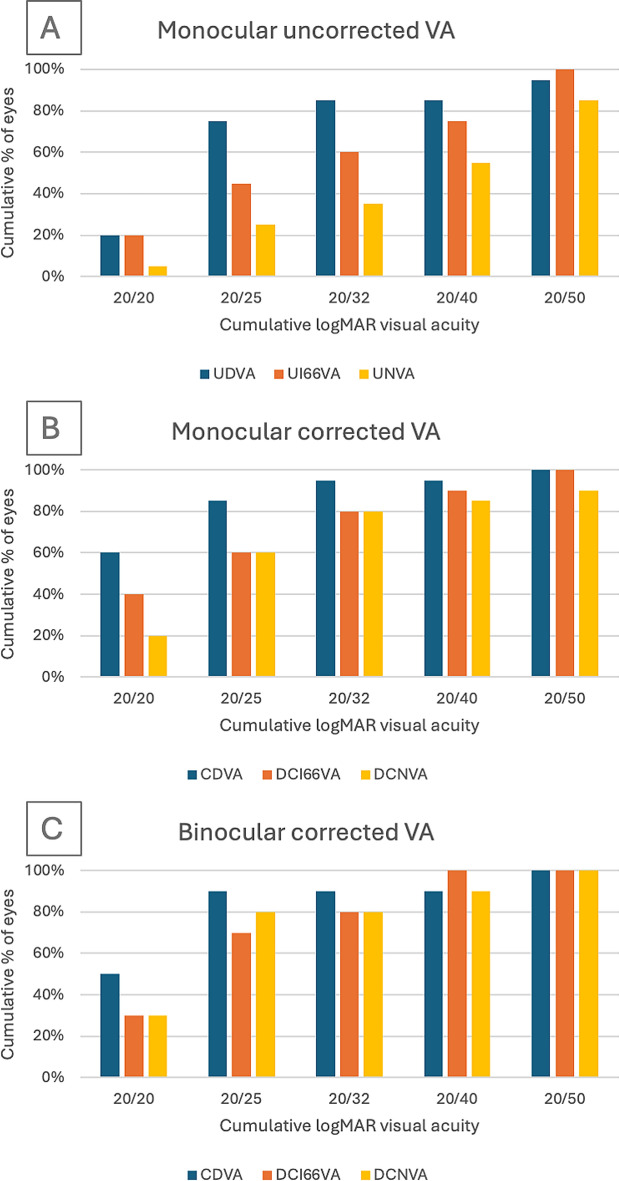
**A–C** Distribution of postoperative monocular and binocular cumulative visual outcomes. VA, visual acuity; UDVA, uncorrected distance visual acuity; UI66VA, uncorrected intermediate (66 cm) visual acuity; UNVA, uncorrected near visual acuity; DCVA, corrected distance visual acuity; DCI66VA, distance-corrected intermediate (66 cm) visual acuity; DCNVA, distance-corrected near visual acuity

Monocular UI66VA and DCI66VA were 0.13 ± 0.16 and 0.14 ± 0.06 logMAR, respectively, while binocular UI66VA and DCI66VA reached 0.09 ± 0.13 and 0.10 ± 0.15 logMAR. (Fig. 2).

The mean DCVA in the dominant eye at 3 months was 0.06 ± 0.13. (Supplemental Fig. 1, 2).

### Defocus curve

**Fig. 3:**
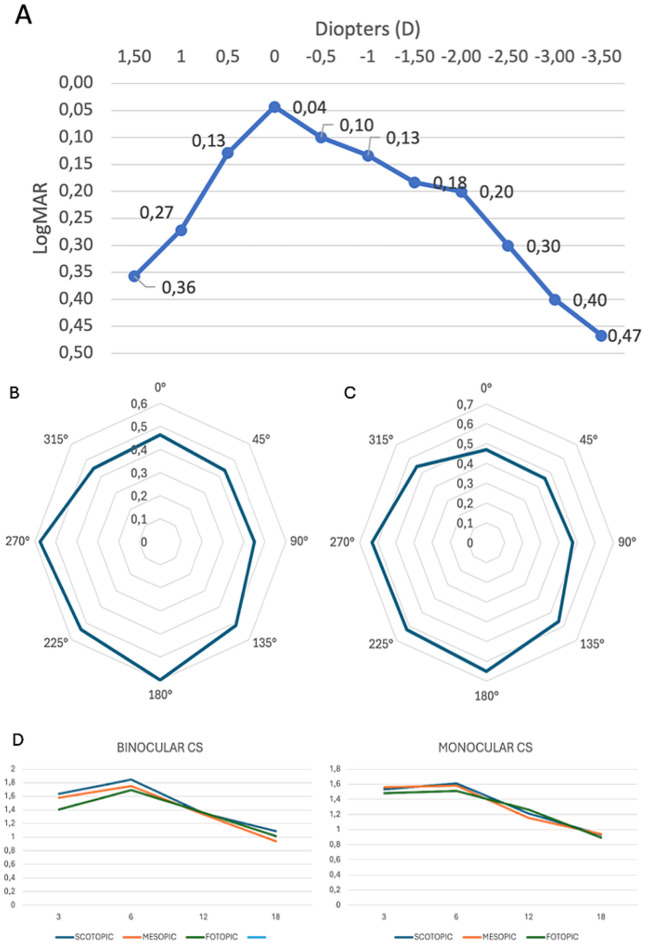
A = Binocular defocus curve. X-axis: defocus lenses added to DCVA; Y-axis: mean visual acuity achieved in logMAR (logarithm of the minimum angle of resolution); B, Halo visual representation in binocular conditions; C** = Ha**lo visual representation in monocular conditions; D, Monocular and binocular contrast sensitivity (CS)

Mean visual acuities were better than 0.13 logMAR between + 0.50 D and –1.00 D. (Fig. 3) Functional visual acuities were better than 0.20 logMAR across the extended range from + 0.50 D to – 2.00 D.

### Objective and subjective quality of vision

Mean total ocular RMS decreased from 2.15 ± 1.98 to 1.09 ± 0.77 (*p* = 0.09). Mean RMS and PSF Strehl ratio at 3 mm of pupil diameter were 0.51 ± 0.31 and 0.30 ± 0.20, and 0.85 ± 0.43 and 0.25 ± 0.16 at 4 mm, respectively.

Across all the tested lighting conditions, the highest monocular CS occurred under scotopic condition at 6 cpd (1.61 log units). Sensitivity declined at higher spatial frequencies, with the lowest values in mesopic condition at 18 cpd (0.93 log units). CS were higher binocularly. Figure 3 shows CS curves and halometry results. NEI-RQL-42 scores were highest for “suboptimal correction” and “activity limitation” (Table 2).

**Table 2:** Subjective quality of vision by means of NEI- RQL-42 scores

Subscale evaluated	Mean ± SD
Clarity	68.54 ± 32.17
Expectations	42.50 ± 37.36
Near vision	70.20 ± 22.99
Far vision	74.67 ± 19.51
Diurnal fluctuation	66.66 ± 32.98
Activity limitation	90.00 ± 21.29
Glare	63.75 ± 27.92
Symptoms	66.78 ± 22.29
Dependence on correction	62.49 ± 26.51
Worry	46.25 ± 36.35
Suboptimal correction	98.75 ± 3.95
Appearance	77.99 ± 28.99
Satisfaction with correction	68.00 ± 21.50

## Discussion

This prospective pilot study evaluated the short-term visual performance of bilateral implantation of the AcrySof IQ Vivity non-diffractive EDOF IOL in carefully selected patients with stable early-stage KC and cataract. The main finding was that this approach was associated with favourable distance and intermediate visual outcomes, functional near vision, acceptable optical quality, and high patient-reported satisfaction at 3 months.

These preliminary findings suggest that stable early-stage KC should not be regarded as an absolute contraindication to non-diffractive EDOF IOL implantation, provided that patient selection is rigorous and preoperative counselling is appropriate.

This interpretation should be limited to the specific population included in the present study. All patients had stable mild bilateral KC, no corneal scarring, preserved historical corrected visual potential, and no individual ABCD parameter greater than grade 1 according to the MS-39 software-derived ABCD output. Therefore, these results should not be extrapolated to eyes with moderate or advanced KC, progressive disease, visually significant corneal scarring, or poor corrected visual potential. In this setting, the key determinant of success is likely not the diagnosis of KC itself, but the degree of corneal regularity, stability, and preserved optical potential.

The primary endpoint of the study was monocular DCVA in the dominant eye at 3 months. Mean postoperative dominant-eye DCVA was 0.06 ± 0.13 logMAR, corresponding approximately to Snellen 20/25. This result indicates that good corrected distance visual acuity can be achieved after implantation of a non-diffractive EDOF IOL in eyes with stable early-stage KC. However, the improvement in visual acuity should not be attributed exclusively to the IOL design, as cataract removal itself is an important contributor to postoperative visual recovery.

The present findings can be interpreted in the context of the limited literature on presbyopia-correcting IOLs in KC eyes. Mahmood and Alsaati [34] reported favourable satisfaction and spectacle-independence outcomes after diffractive trifocal IOL implantation in eyes with stable subclinical and forme fruste KC, with high satisfaction despite the occurrence of haloes and night-driving difficulty in some patients. Farideh et al. [33] also reported favourable postoperative visual acuity after diffractive trifocal IOL implantation in stable subclinical and forme fruste KC, with a mean postoperative DCVA of 0.10 logMAR at 3 months. Although the mean DCVA in the present cohort was numerically favourable, direct comparison should be avoided because of differences in patient selection, IOL platform, KC severity, and methodology. The difference between diffractive trifocal and non-diffractive EDOF optics is clinically relevant. Diffractive trifocal IOLs may provide stronger near vision, whereas non-diffractive EDOF technology is expected to provide a smoother range of vision, particularly across distance and intermediate vergences, with potentially lower optical disturbance. In the present study, the defocus curve was consistent with the expected behaviour of a non-diffractive EDOF IOL, showing useful visual acuity from distance through intermediate vergences, with more limited but functional near vision. This profile differs from the more segmented defocus pattern typically observed with trifocal IOLs, which may perform better at higher near additions but at the cost of light splitting and potentially greater photic phenomena. Therefore, the present results should not be interpreted as evidence of full-range near performance comparable to trifocal technology, but rather as support for good distance and intermediate function with acceptable near vision in a selected KC population.

At intermediate distance, the results were also consistent with those reported in non-KC eyes implanted with the same IOL platform. Kohnen et al. [35] reported distance-corrected intermediate visual acuity values around 0.14–0.15 logMAR at 66–80 cm, while Barberá-Loustaunau et al. [36] reported a monocular DCIVA of approximately 0.14 logMAR after Vivity implantation. The comparable intermediate performance observed in the present cohort suggests that mild stable KC, when associated with preserved central optical quality, may not preclude the functional benefit expected from a non-diffractive EDOF design.

Near vision was functional but more limited. This is consistent with the optical profile of the Vivity IOL, which was designed to extend the range of vision rather than provide a trifocal near focus. Better binocular near outcomes have been reported in non-KC cohorts, particularly when mini-monovision was intentionally targeted [37]. In contrast, the present study did not frame the procedure as refractive cataract surgery and did not target mini-monovision as a strategy to maximize near vision. This distinction is important when interpreting near visual acuity and spectacle independence. Furthermore, the mild postoperative hyperopic tendency may also have contributed to the relatively limited near performance.

Although 75% of eyes received toric IOLs, postoperative mean residual cylinder remained relatively elevated. This finding is clinically plausible in KC. Manifest refractive astigmatism in KC eyes reflects not only regular corneal toricity, but also irregular astigmatism, coma, and coma-like aberrations that cannot be corrected by a toric IOL. In the present study, toric IOL implantation was considered only when the astigmatic pattern was sufficiently regular and reproducible. Residual cylinder should therefore be interpreted in the context of the underlying ectatic corneal optics rather than as a simple failure of toric correction.

Optical quality outcomes also require careful interpretation. Total ocular RMS numerically decreased from 2.15 ± 1.98 µm preoperatively to 1.09 ± 0.77 µm postoperatively, but this change did not reach statistical significance. Moreover, the reduction in may partly reflect cataract removal rather than the effect of the IOL itself. Postoperative higher-order RMS values remained higher than those reported in non-KC eyes implanted with the same IOL platform, which is expected given the presence of corneal irregularity. Wanten et al. [38] reported lower HOA values in non-KC eyes implanted with non-diffractive EDOF IOLs, supporting the concept that corneal pathology remains a major determinant of optical quality even after successful cataract surgery.

CS outcomes were generally favourable. Kohnen et al. [35] reported photopic and mesopic CS values after Vivity implantation in non-KC eyes that were broadly comparable to those observed in the present cohort. Wanten et al. [38] also found only limited differences in CS between modern non-diffractive EDOF platforms, suggesting that contrast performance may be relatively preserved with this category of IOL. In the present study, CS peaked under scotopic conditions at 6 cycles per degree. Several mechanisms may contribute to this finding, including pupil-dependent use of the peripheral optic and the absence of diffractive light splitting. However, this interpretation remains speculative because pupil diameter was not measured during CS testing. Therefore, the scotopic CS findings should be interpreted cautiously.

The theoretical advantage of a non-diffractive EDOF IOL in early KC lies in its optical conservatism. Vivity uses a central wavefront-shaping design to extend depth of focus without a diffractive light-splitting profile. This may be preferable in eyes with mild corneal irregularity, where preserving contrast and minimizing additional optical phenomena are important. Other EDOF platforms, such as Mini WELL, extend depth of focus through different optical principles, including the induction of opposite spherical aberrations. This may provide stronger near performance in some settings but may also increase optical complexity. The present study was not designed to compare EDOF platforms, but the findings suggest that the relatively conservative optical profile of Vivity may be compatible with selected early-stage KC eyes.

The patient-reported outcomes further support the clinical feasibility of this approach. High satisfaction in the present cohort is consistent with previous reports of Vivity implantation in non-KC populations. Barberá-Loustaunau et al. [36] reported high rates of satisfaction and spectacle independence after clear lens exchange with the Vivity IOL, with limited photic phenomena. Kohnen et al. [35] similarly reported favourable patient-reported outcomes after bilateral Vivity implantation, with halo and glare not significantly affecting overall satisfaction. Although these studies were performed in non-KC eyes, they provide useful context for interpreting the patient-reported outcomes observed in the present cohort.

The present findings are also consistent with the growing interest in non-diffractive EDOF IOL implantation in eyes with selected ocular comorbidities or non-standard optical conditions. Favourable outcomes have been reported in eyes with mild glaucoma, low-grade epiretinal membrane, early age-related macular degeneration, and previous myopic LASIK [13–15, 39]. These conditions differ substantially from KC, but they share the concern that premium IOL implantation may reduce visual quality or increase dissatisfaction. Together with the present results, these data suggest that careful assessment of optical quality, visual potential, and patient expectations may be more relevant than the diagnostic label alone.

The conservative management of residual refractive error is an important aspect of this study. Although a non-diffractive EDOF IOL was implanted, surgery was not framed or managed as standard refractive cataract surgery. Patients were counselled preoperatively that KC could limit refractive predictability and that mild residual refractive error might persist after surgery. In symptomatic patients, spectacle correction was considered the first-line management option. Corneal refractive enhancement was not included in the management protocol because of the underlying ectatic disease.

This study has several limitations. First, it was a non-comparative pilot study with a small sample size of 10 patients and 20 eyes, limiting statistical power and generalisability. Second, follow-up was limited to 3 months. Longer follow-up is required to assess refractive stability, toric IOL rotational stability, KC progression, late-onset photic phenomena, and durability of patient satisfaction. Third, the absence of a control group prevents direct attribution of the observed outcomes to the non-diffractive EDOF design. Fourth, pupil diameter was not measured during contrast sensitivity testing, limiting interpretation of the scotopic contrast sensitivity findings. Fifth, although ABCD grading was available as an integrated output of the MS-39 software, the original Belin ABCD system was described using Scheimpflug tomography. Accordingly, MS-39-derived ABCD staging should not be considered automatically interchangeable with Pentacam-derived ABCD staging. For this reason, in the present study, ABCD grading was used only as one component of KC staging, together with clinical examination, absence of corneal scarring, good historical corrected visual acuity, and documented tomographic stability.

In summary, this prospective pilot study provides preliminary evidence that bilateral implantation of a non-diffractive EDOF IOL may be clinically feasible in highly selected patients with stable early-stage KC and cataract. The procedure was associated with favourable short-term distance and intermediate visual outcomes, functional near vision, acceptable contrast sensitivity, and high patient satisfaction. These findings are hypothesis-generating and require confirmation in larger comparative studies with longer follow-up and more diverse KC populations.

## Conclusions

In carefully selected patients with stable early-stage KC and cataract, bilateral implantation of the AcrySof IQ Vivity non-diffractive EDOF IOL was associated with favourable short-term visual and patient-reported outcomes. The results suggest that stable early-stage KC may not represent an absolute contraindication to non-diffractive EDOF IOL implantation. However, given the small sample size, non-comparative design, and short follow-up, these preliminary findings should be confirmed in larger prospective comparative studies before broader clinical recommendations can be made.

## Supplementary Information

Below is the link to the electronic supplementary material.

Supplementary Material 1.

Supplementary Material 2.

Supplementary Material 3.

## Data Availability

No datasets were generated or analysed during the current study.
