# Supplementary material for: Visual performance of a non-diffractive extended depth of focus IOL in stable early-stage Keratoconus: prospective pilot study
Source: Int Ophthalmol. 2026 Aug 4;46(1):323. doi: 10.1007/s10792-026-04186-5 (PMC13437671; doi:10.1007/s10792-026-04186-5)
Supplement: Supplementary file 1 [file 10792_2026_4186_MOESM3_ESM.docx]

**Supplemental Figure 1 A-D**. Post operative differences between monocular corrected and uncorrected visual acuity at 4 m, 66 cm and 40 cm. UDVA = uncorrected distance visual acuity; UI66VA = uncorrected intermediate (66 cm) visual acuity; UNVA = uncorrected near visual acuity; DCVA = corrected distance visual acuity; DCI66VA = distance-corrected intermediate (66 cm) visual acuity; DCNVA = distance-corrected near visual acuity.

**Supplemental Figure 2 A-D**. Post operative differences between binocular corrected and uncorrected visual acuity at 4 m, 66 cm and 40 cm. UDVA = uncorrected distance visual acuity; UI66VA = uncorrected intermediate (66 cm) visual acuity; UNVA = uncorrected near visual acuity; DCVA = corrected distance visual acuity; DCI66VA = distance-corrected intermediate (66 cm) visual acuity; DCNVA = distance-corrected near visual acuity.
